# Roll-to-roll fabrication of superhydrophobic pads covered with nanofur for the efficient clean-up of oil spills

**DOI:** 10.3762/bjnano.13.102

**Published:** 2022-10-31

**Authors:** Patrick Weiser, Robin Kietz, Marc Schneider, Matthias Worgull, Hendrik Hölscher

**Affiliations:** 1 Institute of Microstructure Technology (IMT), Karlsruhe Institute of Technology (KIT), P.O. box 36 40, 76021 Karlsruhe, Germany; 2 Karlsruhe Nano Micro Facility for information-driven Material Structuring and Characterization (KNMFi), Karlsruhe Institute of Technology (KIT), Hermann-von-Helmholtz-Platz 1, 76344 Eggenstein-Leopoldshafen, Germany

**Keywords:** hot embossing, lotus effect, nanofur, nanopads, oil spill cleanup, oil water separation, roll-to-roll, R2R, superhydrophobicity

## Abstract

Superhydrophobic surfaces, which self-clean through rinsing with water, have gained significant importance during the last decades. A method to fabricate such a surface featuring the lotus effect, solely through structuring, is hot pulling of a polymer surface. This technique provides the so-called nanofur, which consists of a polymer surface densely covered with a polymeric fur of extremely thin hair-like structures. Here, we present a continuous roll-to-roll process for the fabrication of a thin polymeric film covered with nanofur from polypropylene. Our process enables structuring of large areas of the order of square meters using industry standard machinery. This opens up many possible applications for nanofur that could previously not be realized because of the limitations of conventional hot embossing regarding structurable area. The structured film is subsequently processed into an exemplary product, that is, so-called nanopads; polymeric sandwiches of polypropylene film covered with nanofur and filled with an oil-absorbing material. These are well-suited for the cleanup of small oil spills.

## Introduction

Self-cleaning surfaces utilizing the famous lotus effect have gained significant importance during the last twenty-five years [1]. Prominent examples include wall colors that let dust and soil drop off when it rains [2], hydrophobic coatings for glass surfaces (e.g., cameras at toll stations or windshields for better visibility [3]), anti-graffiti paints [4], as well as textiles that repel unwanted dirt [5–6]. The lotus effect is commonly achieved by hierarchical nano- and micro-structuring of surfaces made from materials with low surface energy leading to very high contact angles (above 150°). This strategy is inspired by the lotus leaf [1] but can be found on many other surfaces in nature, too [7].

Multiple techniques exist to prepare self-cleaning surfaces. Direct laser writing and electron beam lithography have been employed successfully to create superhydrophobic surfaces. However, due to low writing speeds these approaches are not viable for surface areas larger than a few square millimeters [8–9]. Various (soft) lithography techniques have been employed to create superhydrophobic surfaces; however, these generally rely on copying surface information from a master (e.g., a lotus leaf) [9–10] and are therefore often limited in size. Superhydrophobic surfaces could also be prepared using various dry/wet etching techniques including electrochemical HF etching, stain etching, metal-assisted etching, and reactive ion etching [9,11]. So-called “nanograss” or “black silicon” is a surface modification of silicon where the surface is covered with millions of tiny needle-like structures with high aspect ratio. These needles render superhydrophobic properties to the surface. Such surfaces can either be prepared using a RIE process [12] or a laser-assisted etching process described by Mazur et al. [13], which requires expensive silicon wafers as substrate as well as a femtosecond laser source [11,14]. All mentioned processes require potentially hazardous chemicals, chambers creating a suitable process environment, or extensive and costly operations to form superhydrophobic surfaces. Hence, they are not particularly well suited for upscaling.

A rather cost-effective and, therefore, widely used method for obtaining superhydrophobic surfaces is the use of silica-based films, which can be applied by dipping the object in gel or via aerosol spray [15]. While providing excellent superhyrophobicity and an easy application even on very large surfaces, these films are not particularly durable. Other options for chemically treated superhydrophobic surfaces, such as the use of fluorinated silanes, fluoropolymer coatings, and carbon nanotubes, exist, but are either rather costly to apply and/or potentially harmful to the environment.

A much simpler and cheaper option is the fabrication of polymeric nanofur [16]. Its surface is covered with many tiny, hair-like structures and has a high potential for up-scaling because it can be produced with minimal, very simple and cost-effective tools and molds [17]. On the lab scale, the fabrication of nanofur can be easily achieved with sand-blasted steel plates serving as form inserts in a hot embossing machine [18]. However, in this case the fabrication is limited to areas of some square centimeters. These sizes are not sufficient for commercial applications.

To overcome this hurdle and to allow for the cost-effective fabrication of thin polymeric nanofur, we developed a continuous roll-to-roll (R2R) process. For that, we combine classical film extrusion with roll-to-roll structuring [2,19–22]. The overall process relies only on tools frequently used in industry, thereby enabling the cost-effective upscaling of nanofur fabrication for commercial applications.

Furthermore, we demonstrate the subsequent processing of the thin polymeric nanofur into an exemplary product, namely so-called nanopads. These are an aid for the efficient cleaning of oil spills due to the efficient oil–water separation ability of nanofur [23]. The polymeric pads are 48 mm in diameter and filled with an oil-absorbing material. On the outside, they are covered with superhydrophobic and oleophilic nanofur, which repels water and attracts oil at the same time. In order to utilize this feature in an efficient way and to allow for continuous oil absorption, the polymer foils are equipped with tiny holes through which oil is absorbed and finally saved in the embedded oil-absorbing material.

## Results and Discussion

This chapter is written in two parts. The first one describes the fabrication process for large area nanofur, and the second one deals with the production of an exemplary product from that nanofur. This split is done for better clarity since these are essentially two separate processes.

### Roll-to-roll fabrication of nanofur

Nanofur is a polymeric surface covered with a dense fur of very thin and long hair-like structures [16]. This arrangement causes a significant increase of the contact angle of water droplets. The fractal structure minimizes contact area as well as adhesion forces between surface and water droplet, thereby equipping the surface with self-cleaning properties equivalent to the lotus effect [24]. Additionally, the high increase of surface area increases the spreading of non-polar liquids. Therefore, a nanofur surface separates oil and water efficiently [16–17,23].

In the abovementioned studies, the fabrication of nanofur was based on a hot-pulling process, which is commonly realized in a hot-embossing machine utilizing sandblasted steel-plates as mold inserts. It is an interesting feature of this procedure that a surface with nanostructures is realized without elaborate and costly lithography. Nanofur can be hot-pulled from several types of polymers [16]. Nonetheless, the surface area per process step is limited to some square centimeters since hot embossing is a serial process [18]. In the view of commercial applications, however, a continuous process is highly desirable.

**Figure 1:**
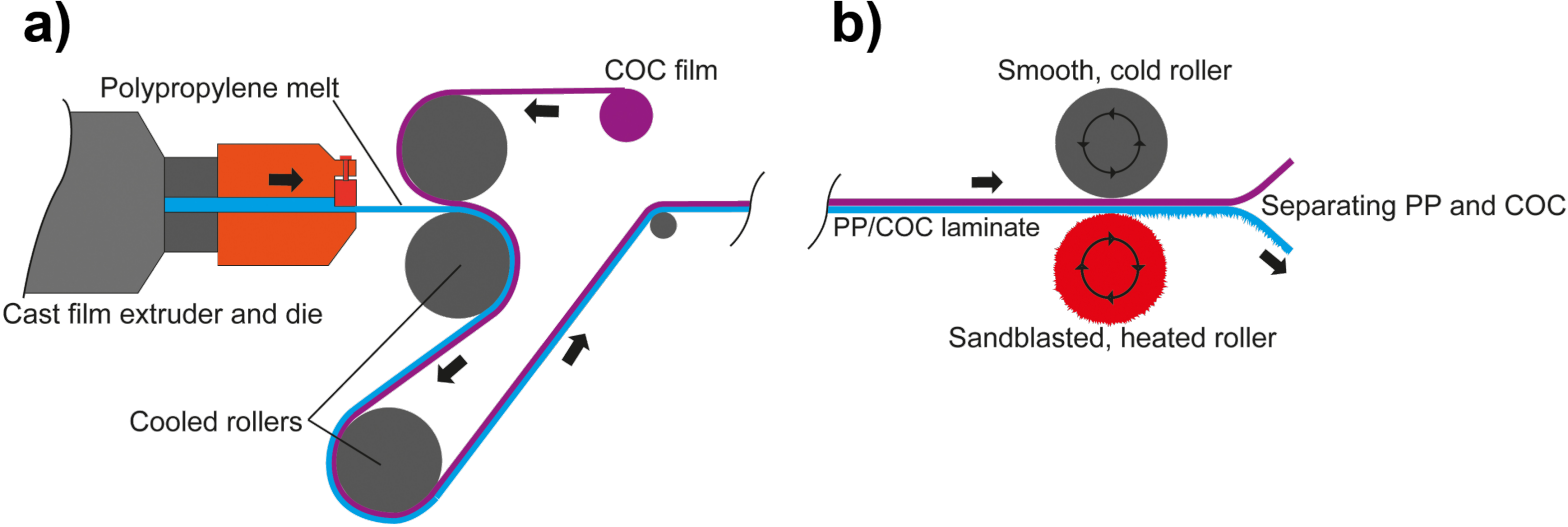
Schematic showing the roll-to-roll fabriction of a thin nanofur film by the example of PP and COC. (a) Coextrusion of PP on the COC support layer. The PP is extruded through a cast film die and laminated onto a previously extruded COC film in the calender. The PP and COC films stick strongly together but do not mix. (b) Hot pulling of nanofur from the PP film with a sandblasted roller. The COC film serves as a support layer keeping the PP from melting completely and thus enables transport through the calender. The gap size is set slightly below the nominal film thickness so that the films experience minimal embossing forces. Temperature of the sandblasted roller is set slightly above the melting temperature of the polymer. Finaly, the structured PP and the sacrificial support layer of COC can easily be separated in a peeling motion.

As demonstrated here, the continuous fabrication of superhydrophobic nanofur is possible in a roll-to-roll (R2R) process. Figure 1 outlines the two universal process steps. First, a thin polymer foil is extruded on a sacrificial layer (Figure 1a). In a second step, the nanofur is hot-pulled from this extruded film in a R2R machine with a sandblasted roller. Subsequently, the nanofur film can be peeled of the sacrificial layer (Figure 1b). This sacrificial film is needed to provide the suitable process stability during the hot-pulling step. For comparably thick and stable polymer films (thicker than 1 mm) it is also possible to omit this support layer. In the following, however, we focus on thin nanofur films fabricated with a sacrificial layer because these are well-suited for the subsequent processing into nanopads introduced in the introduction and described in more detail in the following section.

We selected polypropylene (PP) as base material for the hot pulling of nanofur for three reasons. First, even its flat surface features a comparable high contact angle (95–100°, depending on the exact type [25–27]), which helps to achieve a high contact angle also for the structured surface. Second, we know from previous studies that it has a comparable wide process window for the hot pulling of nanofur [28]. Third, polypropylene is well-suited to be laminated on films of cyclic olefin copolymer (COC). As shown by Kolew [29], these two polymer types adhere strongly but do not mix during the extrusion process. Hence, the combination of PP and COC is a good match for the fabrication of nanofur with the R2R process sketched in Figure 1.

**Figure 2:**
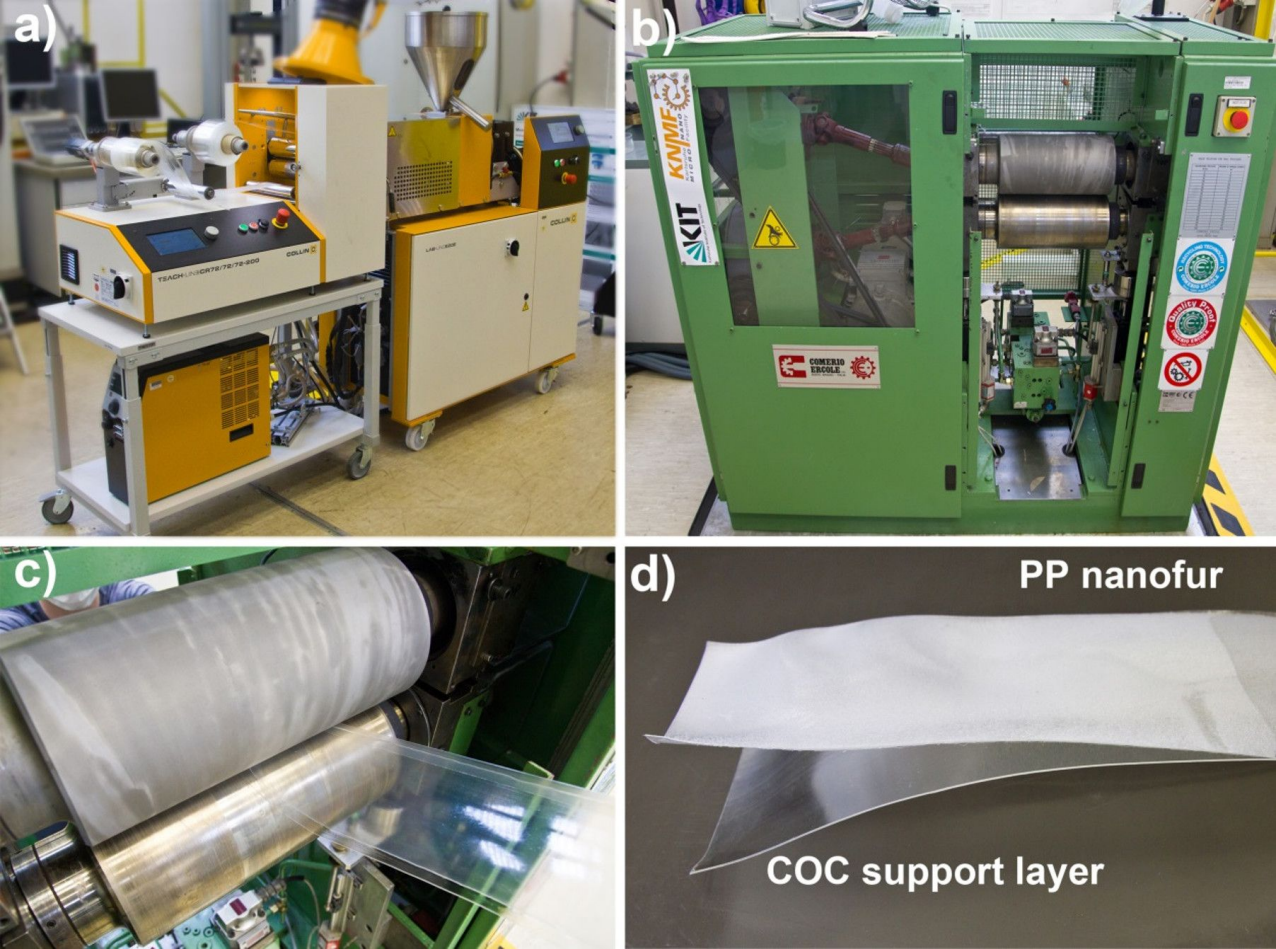
Photos showing the essential fabrication steps of thin nanofur. (a) Extrusion line with rolling unit to co-extrude the thin PP film onto the COC support film. The right roll is the coiled COC film; the left one is the completed PP/COC laminate. (b) Photo of the lab calender utilized to structure the PP film. (c) Close-up of the rollers of the lab calender. In this machine the upper sandblasted roller is heated and gets in contact with the PP side of the laminate. In this way, the nanofur hairs are pulled out of the PP film. The lower roller is smooth and not heated, but presses the film sandwich against the upper sandblasted roller. (d) Finally, PP and COC are separated through peeling.

For that, we extruded a 250 µm thick COC film (TOPAS COC 8007s) in a lab extruder (Collin E20E - E30E) (Figure 2a). Since COC is non-hygroscopic, drying of the granulate is not necessary. The polymer melt was extruded at a temperature of 260 °C at 60 bar. Behind the extruder, there is a calender with smooth, temperature-controlled rollers (Collin CR 72/72/72-200 T) smoothing out and rolling up the film at about 1.3 m/min.

After the preparation of this COC film, which serves as sacrificial support layer, polypropylene is processed into a 150 µm thick film using the same extruder (Figure 2a). During this process, sketched in Figure 1a, the film is laminated onto the COC film resulting in a PP/COC sandwich. In our case this laminate was coiled and subsequently structured in a lab calender (Figure 2b). However, in an upscaled commercial process the structuring might follow the lamination process directly in a combined machinery.

For the structuring of the PP film, schematically shown in Figure 1b, the roller serving as structuring tool was prepared in two steps. First, it was sandblasted with cast iron particles with a diameter of 0.8–1.2 mm and afterwards with white corundum with a particle size of 53–75 µm. This procedure results in a surface topography of craters and edges with finer spikes on top. This sandblasted roller is mounted in a two-roller calender (Figure 2b) and heated (Figure 2c). Best quality nanofur in terms of high contact angles is obtained if the temperature is set slightly above the melting point of the respective PP type (170 °C in our case). For this temperature the viscosity of the polymer melt was sufficient to enable the hot-pulling process. The second roller of the calender was unheated and had a smooth surface. The gap between the rollers was adjusted to be slightly thinner (a few tens of micrometers) than the nominal material thickness. In this way, the laminated films experience only minimal embossing forces during their pass through the calender and the rough sandblasted surface of the heated roller pulls tiny polymer hairs out of the melted surface of the PP film. During this structuring step, the COC layer serves as a support layer enabling the processing of thin nanofur films that would melt entirely and/or stick to the hot roller without the COC support film.

**Figure 3:**
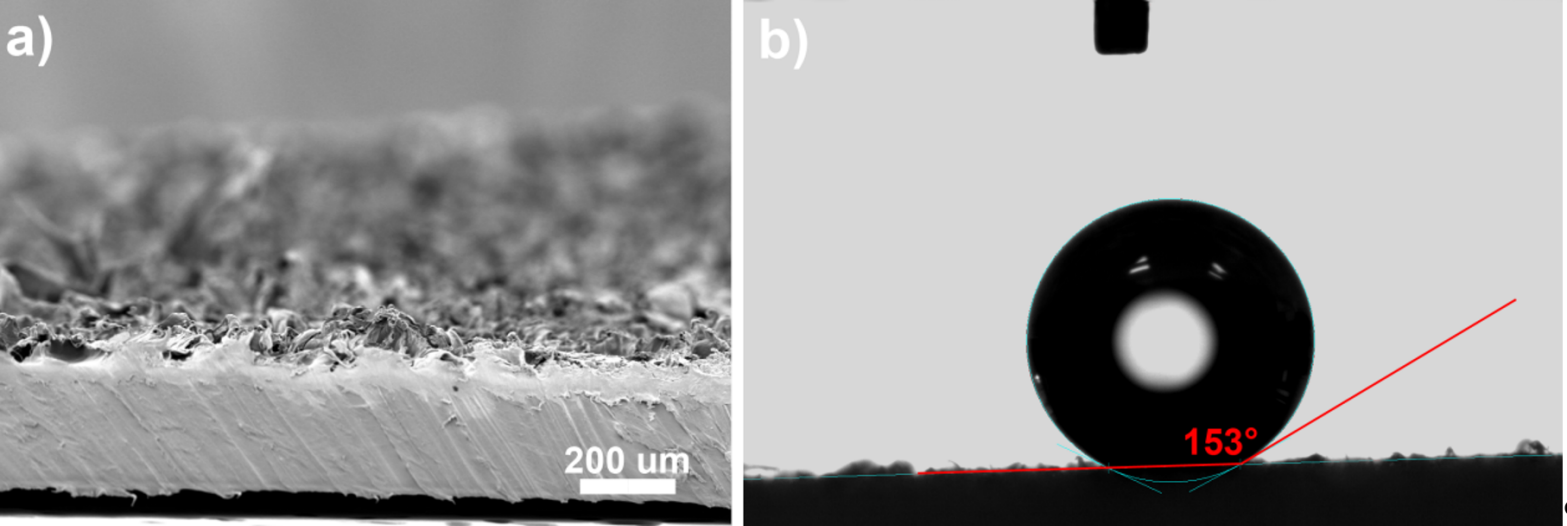
(a) SEM picture of a side cut of a PP nanofur film (view angle 84°). (b) A water droplet of 1 µL on top of the PP nanofur. The contact angle at this spot was 153°.

After structuring, the PP film can be separated from the COC either by hand or, in the case of later envisioned commercial applications, by two winding rollers that separate the two foils with a peeling motion (Figure 2d). The SEM (Zeiss Evo 10) picture in Figure 3a reveals the typical crater-like topography of nanofur with hair on the crater edges [16]. Typical contact angles of water on top of the nanofur are around the super hydrophobicity limit of 150° (see Figure 3b). This makes droplets easily roll off a sample even at minimal tilt (see the video in Supporting Information File 1). Contact angles were measured on a Dataphysics OCA 20 contact angle measurement machine using distilled water in the sessile drop method with a droplet volume of 1 µL. Due to the hydrophobic nature of the surface, drops tend to roll to areas with lower contact angles when performing measurements. This leads to slightly lowered average contact angles. The relatively high measurement uncertainty of contact angle measurements, especially on hydrophobic surfaces, can further distort the measured values [30].

**Figure 4:**
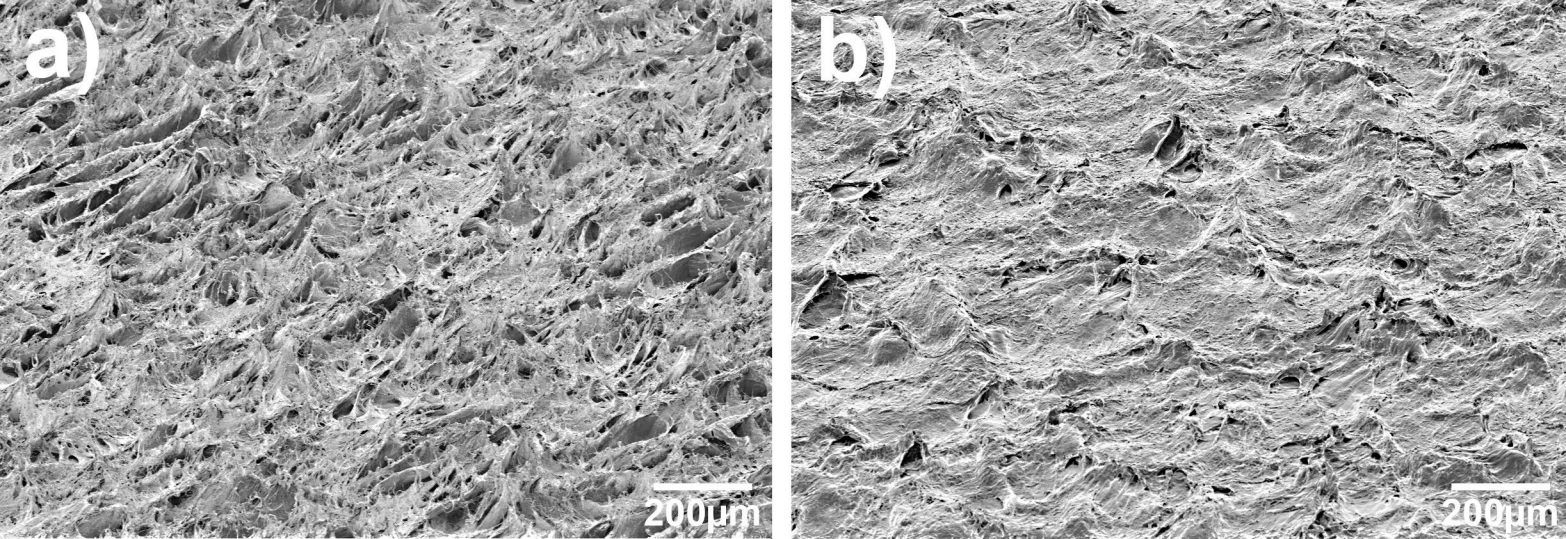
(a) Nanofur produced with a gap size set 50 µm below the nominal material thickness. Many long hairs are pulled out of the material. (b) Nanofur produced with a gap size set 500 µm below nominal material thickness. No hairs are pulled out of the material, the structures look as if they had been flattened. We conclude that a gap size just below the nominal thickness results in good quality nanofur while still structuring the film uniformly, compensating minor thickness variations in the film.

The quality of the nanofur in terms of its hydrophobicity and oil absorption quantity depends on several processing factors including length of the hairs, their density, and their overall uniformity over the whole film area. The most important parameter for high-quality nanofur is the gap size between the upper and lower roller. It should be set to a few tens of micrometers below the nominal material thickness; the contact angle is highest if the roller just touches the surface without applying high embossing forces. In this case it was set to 350 µm (the nominal thickness of the PP/COC laminate was 400 µm). Since the polymer laminate has small variations in thickness, a tradeoff must be made between setting the gap at the optimal thickness for maximum contact angle and setting it below the nominal thickness to compensate thickness variations in the processed film and to structure the film more uniformly. If the gap size is set just at or slightly above the nominal thickness, only the thickest parts of the film are structured. If the gap is too small, no hairs are pulled out of the material, and the resulting structures look as if they had been flattened. Figure 4 shows nanofur produced with a correctly set gap size vs “nanofur” produced with a too narrow gap size.

The hair length can be controlled via the conveying speed of the calender. Slower conveying speeds lead to longer hairs. To a certain extent this can be beneficial because, in general, hairs with high aspect ratio led to high contact angles. However, if the hairs become too long and thin, they might bend under the weight of the water droplets. Furthermore, hair apexes might penetrate water drops. These two effects might slow the speed at which water droplets roll off even if the surface is still superhydrophobic. If the conveying speed is set too high, the hairs are too short and water droplets might touch the surface between the hairs. In this so-called Wenzel state, the droplets are pinned [16]. The video in Supporting Information File 2 shows the effects of hair length on roll-off speed and angle.

The hair density of the nanofur is indirectly controlled via the sandblasting of the structured roller. The optimal roughness of the roller depends also on the actual polymer type. For polypropylene (PP), polyethylene (PE), and polycarbonate (PC), we obtained the best results by sandblasting the roller first with 0.8–1.2 mm cast iron particles and subsequently with white corundum with a particle size of 53–75 µm. For polylactide (PLA), smaller corundum particles with a size of 8–10 µm had to be used for the second sandblasting step to obtain a sufficient hair density [28].

In particular, attention must be paid to the polymer residue left on the structuring roller (Figure 2c). If the residue polymer stays on the hot roller for too long, especially with oxygen present under ambient conditions, it might decompose and inhibit the structuring process. We observed that this effect leads to reduced quality of the nanofur, that is, reduced contact angles. It is therefore important to keep the process running in a continuous fashion as old residues are replaced by newer polymer material. Actually, the contact angle is highest when the roller is newly sandblasted and therefore not covered with any polymer residue. Once the heated and sandblasted roller is covered with residues of polymer the quality of the nanofur reduces slightly.

**Figure 5:**
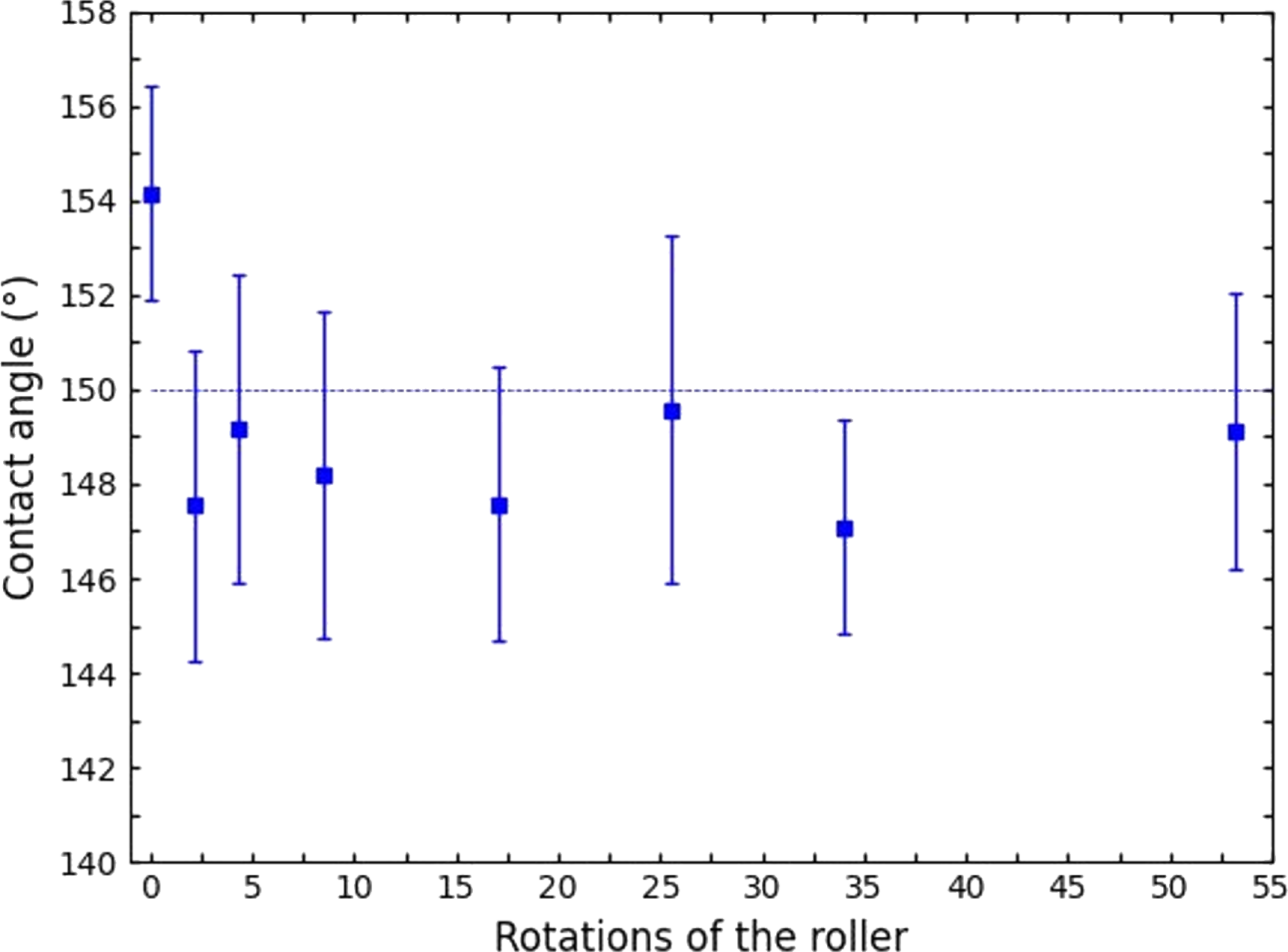
Contact angles measured along a 25 m long foil of nanofur produced in the described roll-to-roll process. A reduction by about 4° is measured when the newly sandblasted roller is first covered in fresh residues. As long as the production process is not interrupted, no further decrease in contact angle occurs.

In order to quantify this effect, we measured the contact angles along a 25 m long film of PP nanofur produced in the fashion described above (see Figure 5). The contact angle is about 154° at first but dropped by 4° after the first turns of the rollers before it became nearly constant with values slightly below 150°. While technically slightly below the limit for superhydrophobicity, these contact angles are still sufficient for almost all typical applications, since the droplets easily roll off the surface even at minimal tilt of the sample. Furthermore the rather large measurement uncertainty of contact angle measurements, especially in the hydrophobic regime, has to be taken into account [30]. To illustrate the superhydrophobic properties of the nanofur, the video in Supporting Information File 1 shows water drops on a polypropylene sheet rolling around as the sheet is tilted. The high contact angle can be seen even with the naked eye.

Since oil absorption is one of the advantageous features of nanofur, we measured its oil absorption capacity. For that, we submerged pieces of the R2R-fabricated nanofur PP film for 20 s in motor oil, let excess oil drip off, and determined the absorbed oil mass by weighing [23]. We used two oils with different viscosity for this analysis. The hydraulic oil TOTAL Azolla ZS 32 had a viscosity and a density of 32 mm^2^/s and 875 kg/m^3^, respectively, and the gear oil TOTAL Carter EP 320 of 320 mm^2^/s and 910 kg/m^3^, respectively. As expected from a previous study [23] the absorption capacity increases with viscosity as the absorbed film thickness increases with this parameter. The PP nanofur absorbed 185 g/m^2^ of Azolla 32 and 561 g/m^2^ of Carter EP320. We conclude that the overall water repellency and oil absorption capacity of the R2R-fabricated nanofur film is in the same range as for nanofur fabricated with the traditional hot-pulling process in a hot-embossing machine [31]. (The videos in Supporting Information File 3 and Supporting Information File 4 show the capability of oil–water separation of nanofur.) Once contaminated with oil, the contact angle with water decreases to below 90°. This makes sense since the contact angle is now essentially measured between the oil film and water. The amount of material that can be fabricated in a R2R process, however, is considerably larger. Furthermore, it is much faster as it is a continuous process. Even with our lab machinery, which is not optimized for speed, relatively large quantities of nanofur can be processed in a matter of minutes (extrusion speed: 1.3 m/min, structuring speed = 2.3 m/min). This, together with the use of machines that are industry standard and the renunciation of complex and therefore costly molds, leads us to believe that our process can fabricate nanofur in a very cost-effective manner, especially on a commercial scale.

**Figure 6:**
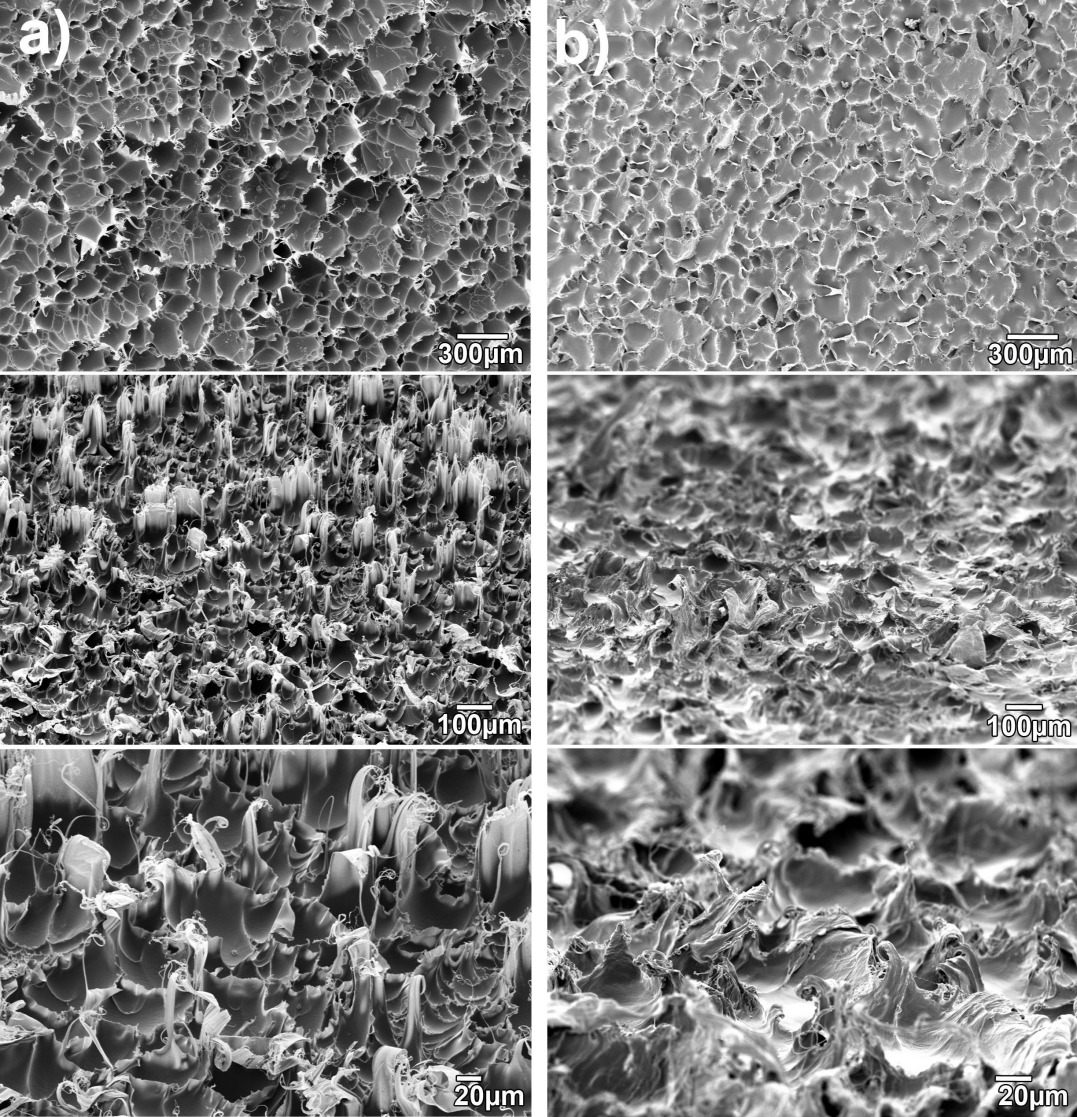
Comparison between nanofur produced by classical hot embossing and R2R nanofur. Even though morphological differences are obvious, the two samples do not differ in function, that is, contact angle. Column (a): Nanofur produced by classical hot embossing. The hairs stand out separately and are relatively straight with a high aspect ratio. Column (b): Nanofur produced by a roll-to-roll process. The topmost pictures are a top view, the others have been taken at a tilt angle of 60°. The structures consist of ridges that culminate in hair-like structures. These ridges can be slightly slanted against the direction in which the polymer traveled through the calender.

While the nanofur produced in this process does not differ significantly in function, that is, contact angle and oil absorption capacity, from nanofur produced by classical hot embossing, the overall structural appearance of the microstructures is different (Figure 6). While the hot-embossed nanofur consists of tiny hairs standing out relatively straight from the surface, R2R nanofur tends to form larger ridges that culminate in hair like tips. Also, since the rollers rotate, these ridges tend to be slightly slanted against the direction in which the polymer traveled through the calender.

### Fabrication of nanopads

It is a remarkable quality of nanofur that it is superhydrophobic and oleophilic at the same time. Nonpolar liquids are immediately absorbed by the fractal polymeric surface while polar liquids are repelled [16]. Consequently, mixtures of oil (nonpolar) and water (polar) are easily separated. A promising application of nanofur is therefore the cleaning of oil spills [23,31]. This behavior can be also utilized for the fabrication of membranes that filter oil out of water (or vice versa) [17]. For that, the nanofur is perforated with small holes, so that oil passes through the polymeric film while water, due to its high contact angle with the surface, cannot penetrate the holes. As shown in the following, we used this property for the development of an exemplary product made of nanofur for the clean-up of oil spills, so-called nanopads.

The standard practice for cleaning up oil spills is scooping the oil film together using barriers, skimming the oil slick, and using separators to clean the remaining water. This method is limited to suitable conditions and, even under optimal circumstances, only recovers about 35% of the spilled oil [32–34]. While somewhat effective for large-scale oil spills, this is unpractical for smaller spills, for example, after car accidents or small tank leaks. Burning the oil is a method often used as well, but requires a certain thickness of the oil film and a low water content in the oil. Furthermore secondary pollution is a problem with burning [34]. Chemical dispersion can be a last-ditch effort to protect sensitive areas from an oil spill but does not separate the oil from the water; many dispersants can also be toxic to the environment [33]. Commonly used sorbents used in the emergency response to oil spills absorb not only oil but also large quantities of water [23,34].

**Figure 7:**
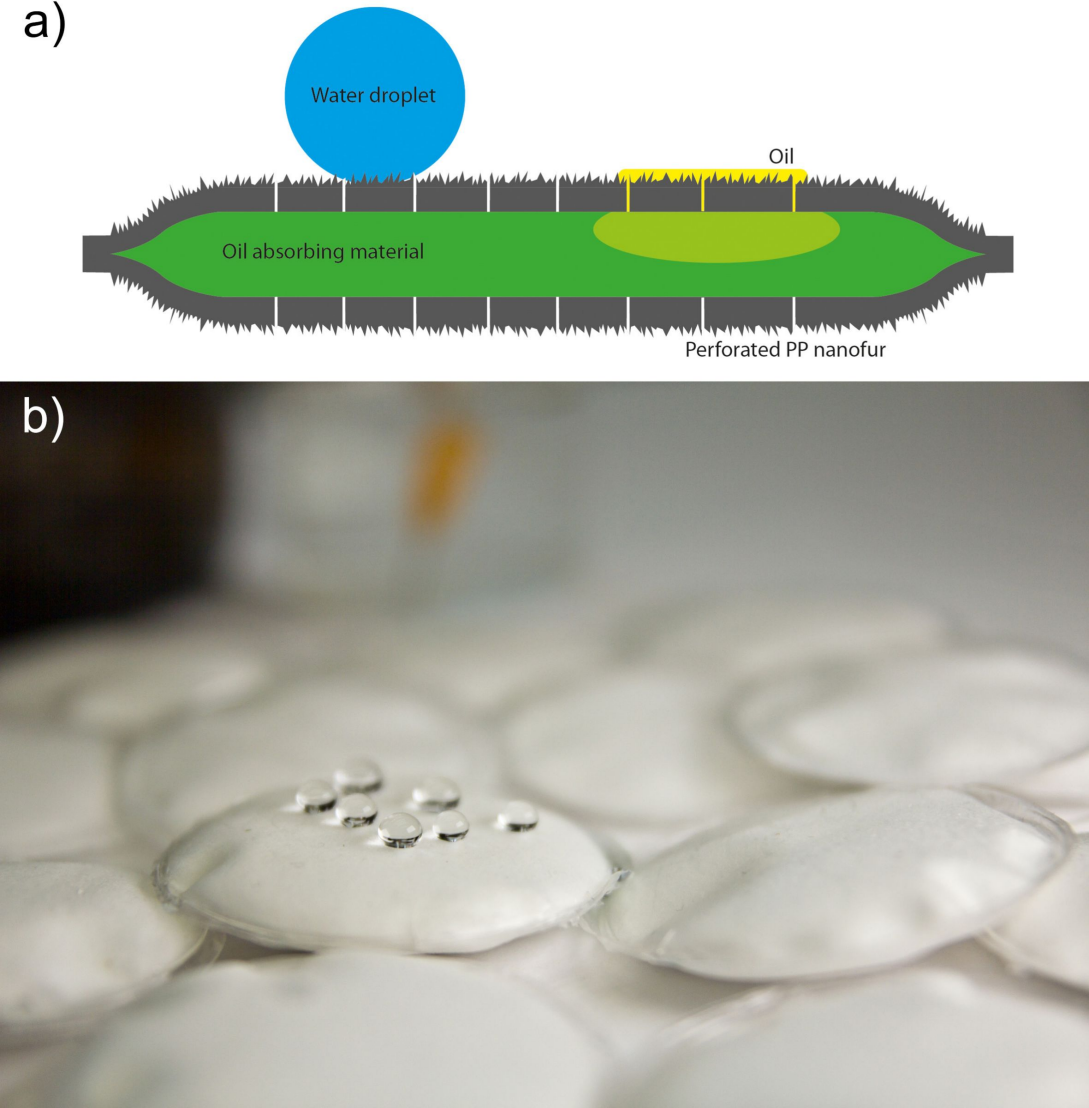
(a) Schematic side view of the nanopads indicating their functionality. Two perforated polypropylene foils, which are covered with nanofur on the outside, enclose an oil-absorbing material. The two foils are heat-sealed at the edge. If the nanopads are dipped in an oil spill, the oil is adsorbed by the nanofur, penetrates into the small holes, and is finally absorbed by the enclosed oil-absorbing material. The small holes are mechanically punched into the nanofur film and have diameters of about 100 µm. (b) Photo of several nanopads (diameter: 48 mm) produced as described in the text. Due to the nanofur cover, the pads are strongly water repellent as demonstrated by the water droplets positioned on the nanopad in the middle of the photo.

The schematic design of the nanofur pads is shown in Figure 7a. The nanopads embed an oil-absorbing material, such as cotton, in a perforated polypropylene film covered in nanofur. The nanofur easily separates oil from water so that the absorbent material can trap the oil.

**Figure 8:**
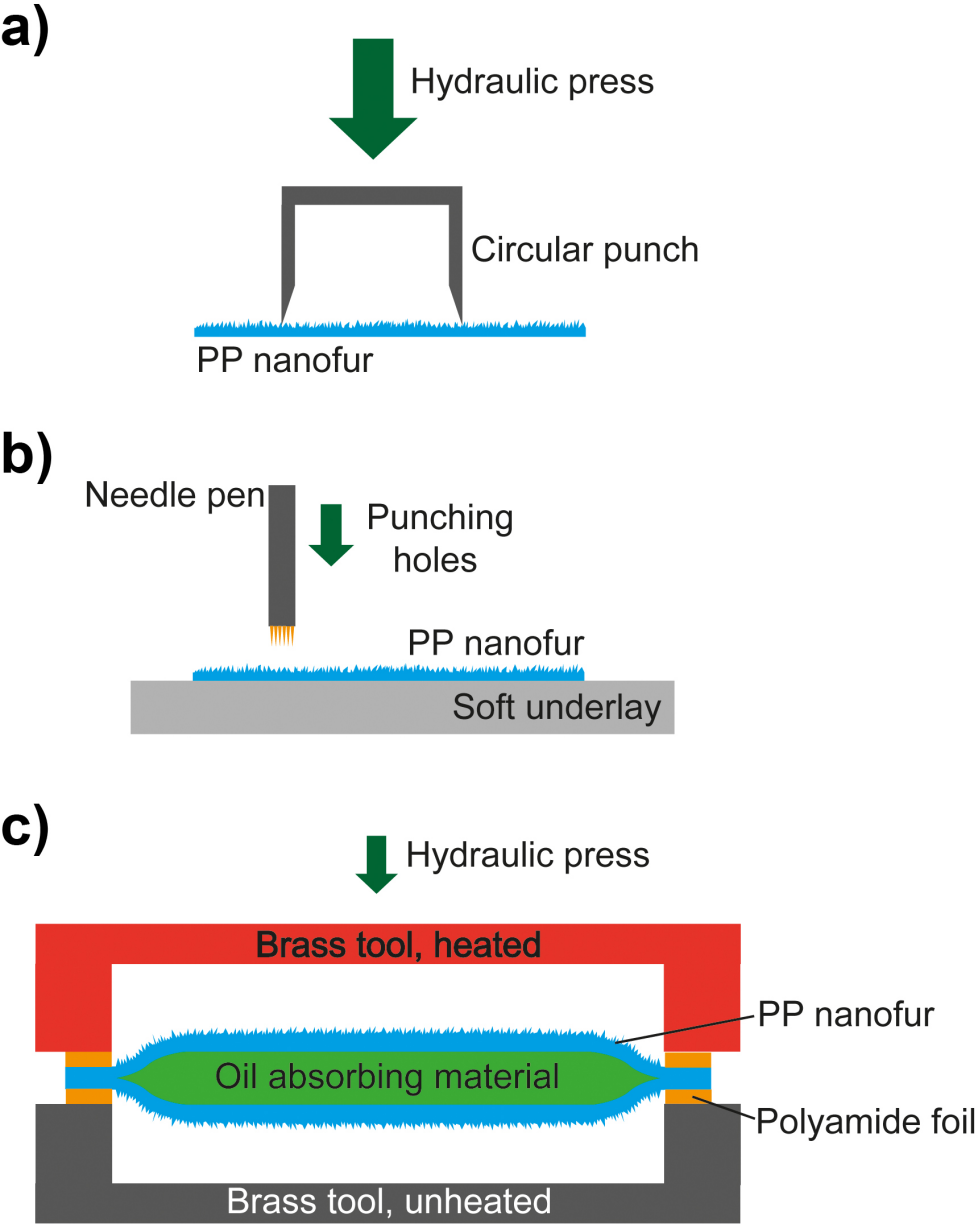
Schematic of the nanopad fabrication steps. (a) First, circular cut-outs are produced using a circular punch. (b) These pieces are perforated by punching holes with a needle pen. A soft underlay guarantees that the needles penetrate the film completely. (c) Two cut-outs are filled with oil-absorbing material, placed between polyimide masks, and heat-welded using a heated brass tool. Finally, the uneven edge is cut off with a circular punch giving the nanopad a round shape.

**Figure 9:**
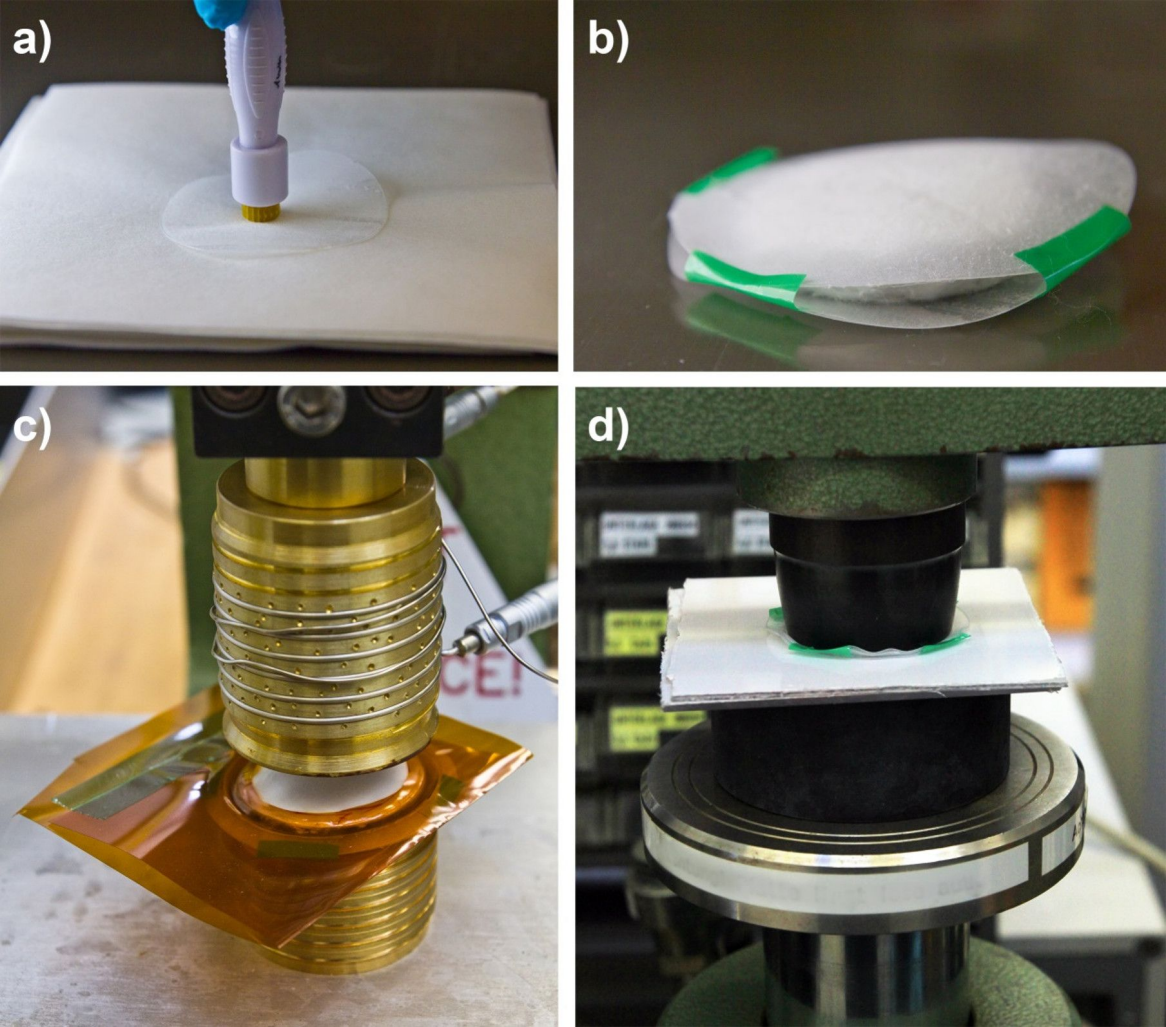
Fabrication of nanopads from polypropylene nanofur film. (a) In the first step, circular pieces are cut from nanofur film with a circular punch. Subsequently, tiny holes are pierced in the nanofur film using a needle pen. The needles have a diameter of about 100 µm. (b) To simplify filling the pads with oil absorbent material, the two halves are held together by scotch tape. (c) Such a preprocessed pad is placed between two polyimide films to avoid cutting through the material while welding. The upper hollow cylinder is heated and then pressed onto the pad for 15 s welding the two halves together. (d) Finally, a circular punch is applied to cut off the excess edge, leading to an evenly round pad.

Figure 7b shows a photo of several manufactured nanopads. The fabrication steps are sketched in Figure 8. Figure 9 shows photos of intermediate fabrication steps. The nanopad fabrication starts with the cutting of circles with a diameter of 60 mm out of the structured PP film (Figure 8a). This is done with a round punch. Afterwards, the film is perforated. This can be done with various types of needles [17]. However, in order to speed up this process step, we used a Derma pen (DRS Dermaroller System) with 42 needles with a diameter of 100 µm each (Figure 8b, Figure 9a). After punching fifteen times, every circular nanofur film had about 600 holes. Afterwards two films are loosely connected with adhesive tape. This step simplifies filling the pad with a predetermined amount of oil-absorbing material (see Figure 9b). For the nanopads analyzed in the following, we used 1.1 g of cosmetic cotton (Rossmann AG). The filled pad is then placed between two polyimide films (DuPont Kapton, 50 µm) just covering the edge to prevent cutting through the material during heat sealing. The welding is carried out in a custom-made tool, consisting of two hollow brass cylinders with a diameter of 48 mm and a wall thickness of 2 mm. The cylinders were heated to 230 °C and pressed on the pad from both sides for about 15 s, welding the two PP films together (see Figure 8c and Figure 9c). The nanofur is destroyed in welded area. Afterwards, the uneven edges are cut off using another circular punch with a diameter of 48 mm (Figure 9d). The resulting pads have a surface area of 2·(2π*r*^2^) = 30.4 cm^2^ covered with nanofur. Due to the hot welding, a 2 mm margin is not covered. The height of the pads is about 10 mm. Extrapolating the shape of the pads as a spheroid, their volume can be calculated as 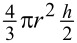 = 10.13 cm^3^.

To measure the quantity of oil the pads can absorb, they were fully submerged in hydraulic oil, transmission fluid, and petrol. The viscosities (at 40 °C) and densities, respectively, of these exemplary oil products are: TOTAL Azolla ZS 32: 32 mm^2^/s and 875 kg/m^3^; TOTAL Carter EP 320: 320 mm^2^/s and 320 kg/m^3^; and Super 95: 0.85 mm^2^/s and 740 kg/m^3^ at 40 °C. These values span a typical range of viscosities for various engine oils and transmission fluids used in everyday tasks and machines [35]. Therefore, such oils are typical products that are likely to cause oil spills, which are supposed to be cleaned up by the nanopads.

**Figure 10:**
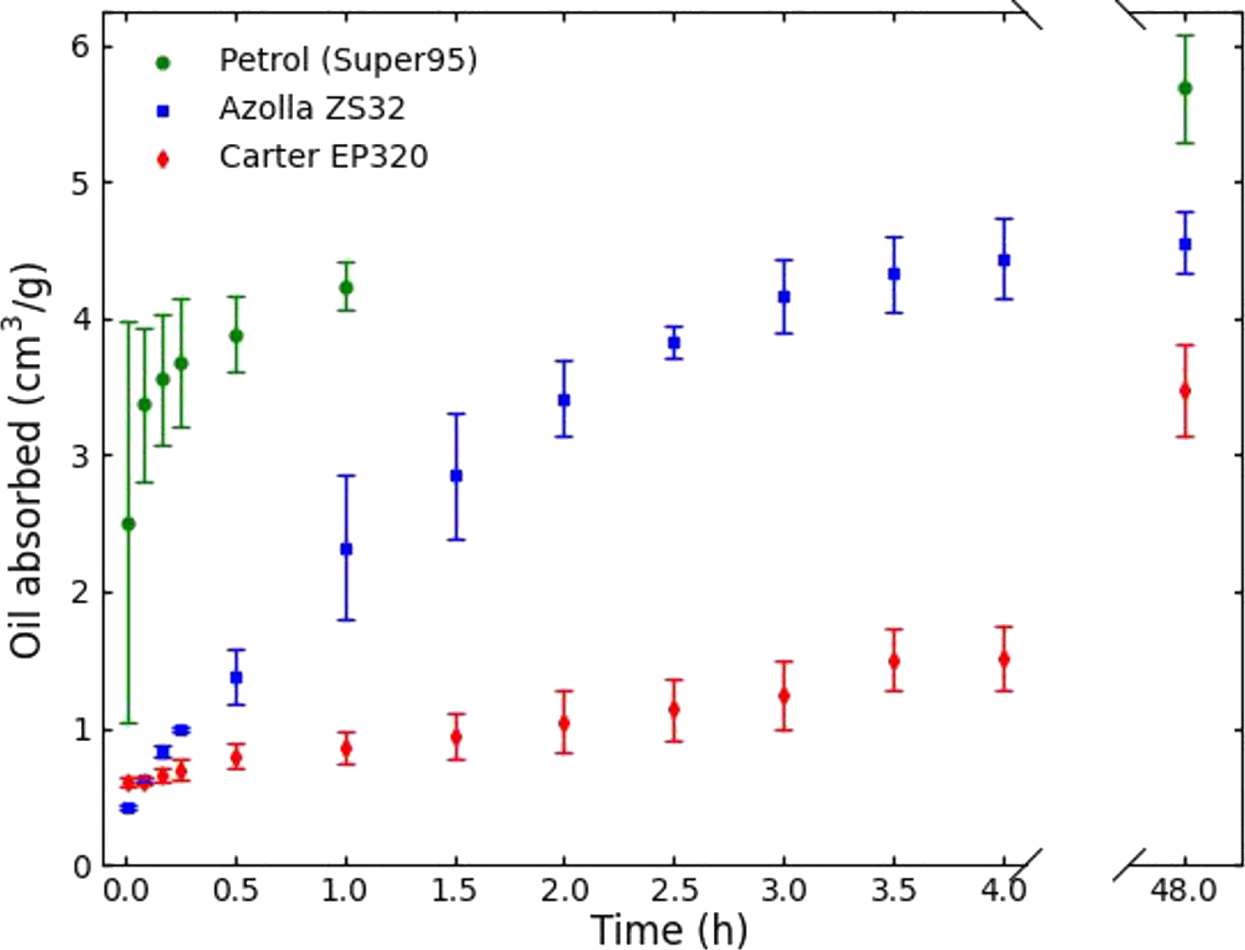
Oil absorption as a function of the time for pads filled with cotton and different oils. The higher the viscosity of the oil, the slower it is absorbed.

After different periods of time, the pads were taken out of the oil and excess oil was allowed to drip off for two minutes. Figure 10 shows the quantity of oil absorbed by the pads after different exposure times. The pads absorb immediately about 0.6 to 0.8 g of oil. This amount is consistent with the quantity of oil that the nanofur surface absorbs. After that, the oil absorption per time decreases because the oil has to penetrate into the nanopads through the punched holes. Therefore, it takes some hours before the pads start to saturate. Finally, additional oil is absorbed very slowly. The last measurement was taken after 48 h to ensure that the pads are saturated completely. The absorbed quantity of oil is similar for all three tested oils, the absorption speed, however, is not. As it can be expected, the viscosity of the oil determines how fast the oil penetrates the nanopads. A lower viscosity leads to faster absorption. As soon as the nanofur is in contact with the oil, an oil film builds up on its surface decreasing the water contact angle to below 90°. Since water and oil cannot mix and the holes are still covered by the oil film, the pads do not absorb any water.

To illustrate the capabilities of the nanopads for oil–water separation and their use in the cleanup of oil spills, the video in Supporting Information File 3 shows a droplet of water–oil emulsion being separated by nanofur. The video in Supporting Information File 4 shows a small drop of colored oil in a jar of water being absorbed completely by a single nanopad.

## Conclusion

We introduced and evaluated a roll-to-roll process to fabricate a large thin polymeric film covered with superhydrophobic and oleophobic nanofur. This process has the potential to be upscaled to industrial standards by utilizing wider rollers in dedicated calenders and, to some extent, by increasing conveying speed and temperature of the roller. The most important parameters for the successful large-scale fabrication of thin nanofur, that is, gap size and roller temperature, have been identified and optimized. The structuring mold is an easy to produce sandblasted steel roller making mold fabrication, reconditioning, or testing of new parameters a straightforward process. Thus, the cost per unit area produced has been significantly reduced; furthermore, it is now possible to obtain large, continuous films covered in polymeric nanofur.

In a further optimization step, it is concievable that the polymer film used as a sacrificial layer, COC in our case, could be reused or recycled. This could be realized either directly, if the surface of the film is not damaged/contaminated or by shredding and extruding a new support film. This sacrificial layer is essential to produce a thin film. It is necessary to find a material that sticks to the polymer the nanofur is to be made of, but can also easily be separated after the structuring step. For films thicker than 1 mm, the mechanical stability of nanofur during hot pulling is sufficient, and the sacrificial layer is not needed. Future research might show how other polymers, especially biodegradable ones, can be hot-pulled in a R2R process. Furthermore, it might be possible to produce thin nanofur without sacrificial layer directly in the extruder from the polymer melt.

The produced thin PP nanofur served as base material to fabricate nanopads. These pads are designed for the clean-up of small oil spills on small waters. Their design might be further optimized for real-life applications. This might include size and shape, as well as the number and diameter of the punched holes. Furthermore, it might be possible to increase absorption speed and amount via the filling or the holes of the nanopads. The filling material itself or its amount might be improved.

Many other applications of thin nanofur, such as new kinds of food packaging and bottles, anti-fouling [36] and antibacterial [37] surfaces, or drag reduction [38] are imaginable. With the low-cost production of large areas of nanofur they seem also financially feasible.

## Supporting Information

File 1 Single droplets of water on a thick sheet of nanofur produced with our roll-to-roll process. The droplets roll off easily at even the slightest tilt of the sample.

File 2 Drops of water on slanted sheets of nanofur. The left sample has longer hairs, the hairs on the right sample are shorter. The video shows that longer hairs tend to pin the drops and, also, that, once the drops roll, they do so much slower than on the samples with short hairs.

File 3 Droplets of oil water emulsion on a piece of nanofur showing oil–water separation.

File 4 A drop of colored oil (TOTAL Carter EP 320 mixed with a small amount of oil paint) is dispensed in a jar of water and then completely absorbed by a single nanopad.
